# A Novel Workflow to Enrich and Isolate Patient-Matched EpCAM*^high^* and EpCAM*^low/negative^* CTCs Enables the Comparative Characterization of the PIK3CA Status in Metastatic Breast Cancer

**DOI:** 10.3390/ijms18091885

**Published:** 2017-08-31

**Authors:** Rita Lampignano, Liwen Yang, Martin H. D. Neumann, André Franken, Tanja Fehm, Dieter Niederacher, Hans Neubauer

**Affiliations:** Department of Obstetrics and Gynecology, Heinrich Heine University of Duesseldorf, Life Science Center, Merowingerplatz 1A, Moorenstr. 5, 40225 Duesseldorf, Germany; rita.lampignano@med.uni-duesseldorf.de (R.L.); liwen.yang@med.uni-duesseldorf.de (L.Y.); neumannmartin1987@gmail.com (M.H.D.N.); andre.franken@med.uni-duesseldorf.de (A.F.); Tanja.Fehm@med.uni-duesseldorf.de (T.F.); Niederac@med.uni-duesseldorf.de (D.N.)

**Keywords:** circulating tumor cell, epithelial cell adhesion molecule*^low/negative^*, CellSearch, Parsortix, CellCelector, phosphatidylinositol 3-kinase catalytic subunit alpha

## Abstract

Circulating tumor cells (CTCs), potential precursors of most epithelial solid tumors, are mainly enriched by epithelial cell adhesion molecule (EpCAM)-dependent technologies. Hence, these approaches may overlook mesenchymal CTCs, considered highly malignant. Our aim was to establish a workflow to enrich and isolate patient-matched EpCAM*^high^* and EpCAM*^low/negative^* CTCs within the same blood samples, and to investigate the phosphatidylinositol 3-kinase catalytic subunit alpha (PIK3CA) mutational status within single CTCs. We sequentially processed metastatic breast cancer (MBC) blood samples via CellSearch^®^ (EpCAM-based) and via Parsortix™ (size-based) systems. After enrichment, cells captured in Parsortix™ cassettes were stained in situ for nuclei, cytokeratins, EpCAM and CD45. Afterwards, sorted cells were isolated via CellCelector™ micromanipulator and their genomes were amplified. Lastly, PIK3CA mutational status was analyzed by combining an amplicon-based approach with Sanger sequencing. In 54% of patients′ blood samples both EpCAM*^high^* and EpCAM*^low/negative^* cells were identified and successfully isolated. High genomic integrity was observed in 8% of amplified genomes of EpCAM*^low/negative^* cells vs. 28% of EpCAM*^high^* cells suggesting an increased apoptosis in the first CTC-subpopulation. Furthermore, PIK3CA hotspot mutations were detected in both EpCAM*^high^* and EpCAM*^low/negative^* CTCs. Our workflow is suitable for single CTC analysis, permitting—for the first time—assessment of the heterogeneity of PIK3CA mutational status within patient-matched EpCAM*^high^* and EpCAM*^low/negative^* CTCs.

## 1. Introduction

Circulating tumor cells (CTCs) are epithelial cells identified in the peripheral bloodstream of patients suffering from cancer and are commonly considered to be responsible for metastases formation in the majority of epithelial solid tumors [1,2]. Since they may differ in genotype and phenotype from the primary tumor, their detection and molecular characterization are of great interest for diagnosis, prognosis and selection of proper medical treatments [3,4]. Despite many technologic improvements, investigating CTCs is still challenging, mainly due to their low numbers in the blood and the necessity of working at single-cell level to overcome intercellular heterogeneity. Currently used CTC-enrichment technologies focus either on the cells’ biological or physical properties [5]. Among them, epithelial cell adhesion molecule (EpCAM)-dependent enrichment techniques are the most widely used with the automated CellSearch^®^ being so far the only Food and Drug Administration (FDA) approved system [6].

It has previously been demonstrated that there are different subpopulations of CTCs ranging in a continuum of phenotypes from the epithelial-like to the mesenchymal-like cells as a result of a process termed epithelial to mesenchymal transition (EMT) [7,8,9]. EMT takes place during tumorigenesis, leading to a loss of a cell’s epithelial features in favor of gaining mesenchymal properties [10,11]. As a consequence, within the same patient, some CTCs may express lower or no levels of EpCAM proteins making them ‘invisible’ for EpCAM-based capturing and detection methods [12,13]. Moreover, it was observed that these tumor cells, which have already initiated EMT, are correlated to worse prognosis—independently on their EpCAM expression—and therefore they might provide additional information about the evolution of the disease [8,9,14,15,16,17,18].

Thus, robust EpCAM-independent detection and isolation approaches are needed to enrich for such CTC subpopulations. Many research groups focused on CTC analysis to investigate their heterogeneity and to stratify metastatic breast cancer (MBC) patients in order to acquire a better cancer-related knowledge and to guide the choice of CTC-based therapeutic treatments.

One of the most studied signaling proteins in breast cancer is the phosphatidylinositol 3-kinase (PI3K), which participates in many significant signaling networks involved in cell growth and survival, and which is frequently altered in many types of human cancer [16,17]. The phosphatidylinositol 3-kinase catalytic subunit alpha (PIK3CA) gene encodes for p110α—the catalytic subunit of PI3K—and is one of the most frequently mutated genes in invasive breast cancer (~26% of the cases), with most mutations clustered in either the helical domain (coded in exon 9) or in the catalytic domain (coded in exon 20), therefore named ‘hotspot’ regions [17]. These mutations were shown to activate PI3K and related pathways, conferring a remarkable selective growth gain to the cell along with in vitro and in vivo tumorigenicity [17,18,19].

However, the prognostic relevance of these mutations has not been clearly figured out, yet. Some recent studies reported improved clinical outcome in patients with luminal breast cancer harboring PIK3CA activating mutations [20,21]. In contrast, a correlation between the existence of PIK3CA mutations and drug resistance was observed, notably in anti-HER2-treatment regimen [22,23,24,25,26]. Early studies on CTCs point towards the presence of PIK3CA hotspot mutations—along with the wild-type form of this gene—in MBC patients [27,28,29,30,31], regardless of the PIK3CA status in the primary tumor [29,30]. Additionally, Markou et al. [29] observed that patients carrying CTCs with mutated PIK3CA have a significant shorter overall survival than those without.

All the abovementioned observations emphasize the necessity of acquiring additional molecular insights into both primary tumors and single CTCs to shed light on the clinical relevance of PIK3CA mutations, and to achieve personalized HER2-targeted therapies.

To the best of our knowledge, the mutational analysis of the PIK3CA within EpCAM*^low/negative^* CTCs has so far not been incorporated, and we believe that this information may actually contribute to understand the anti-HER2 treatment resistance in currently running clinical studies (e.g., DETECT III). Thus, the purpose of our study was to establish a workflow to enrich and isolate both EpCAM*^high^* and EpCAM*^low/negative^* CTCs from the same MBC patient blood sample in order to assess the heterogeneity of the PIK3CA mutational status within the EpCAM*^low/negative^* CTCs in comparison to the EpCAM*^high^* CTC-subpopulation.

For the former, we have recently presented a powerful workflow to enrich for, detect and isolate EpCAM*^high^* CTCs by combining both the CellSearch^®^ system and the CellCelector™ micromanipulator [32]. In parallel, we confirmed the presence of EpCAM*^low/negative^* CTCs in blood discarded after CellSearch^®^ enrichment, further processed with immunomagnetic microbeads targeting proteins expressed on the surface of EpCAM^low/*negative*^ CTCs [13].

**Figure 1:**
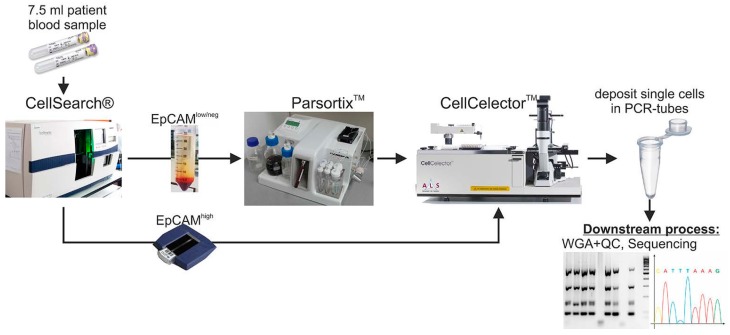
Parsortix™-CellCelector™ workflow. Patients’ blood samples are depleted of EpCAM*^high^* CTCs using CellSearch^®^ and enriched for EpCAM*^low/negative^* cells with the Parsortix™ system. Captured cells are stained for nuclei, cytokeratins, epithelial cell adhesion molecule (EpCAM) and CD45 within the Parsortix™ cartridge and then harvested. Tumor cells are detected and isolated via CellCelector™ micromanipulator into PCR tubes for further molecular characterizations. Inspired by our previous work [32].

However, since this antibody-based approach might still lose a consistent fraction of CTCs with epithelial-mesenchymal plasticity, in the project herein described, we expanded our workflow for CTC isolation and single cell molecular investigation by incorporating an additional label-free CTC enrichment step by using the Parsortix™ system (Figure 1) on blood samples previously depleted for EpCAM*^high^* CTCs. Successively, we isolated patient-matched EpCAM*^high^* and EpCAM*^low/negative^* CTCs and investigated the heterogeneity of their PIK3CA status in all the collected tumor cells via Sanger sequencing.

Our workflow was proved to be suitable for single CTC analysis and has enabled—for the first time—the characterization of the PIK3CA oncogene on patient-matched EpCAM*^high^* and EpCAM*^low/negative^* CTCs.

## 2. Results

### 2.1. A Novel Workflow to Enrich and Isolate Patient-Matched EpCAM^high^ and EpCAM^low/negative^ CTCs

We focused on establishing a robust method that allows the enrichment, isolation and downstream processing of single EpCAM*^low/negative^* CTCs along with the collection of patient-matched EpCAM*^high^* cells. Our workflow starts by processing 7.5 mL blood sample through the CellSearch^®^ system. After depletion of EpCAM*^high^* CTCs, EpCAM-depleted blood is collected and further processed within the Parsortix™ system, representing our workflow′s novel component. Cells captured within this system are labeled with fluorochrome-conjugated antibodies in the Parsortix™ cassette, released and micromanipulated with the CellCelector™ system within 48 h. CTCs are identified based on the following features: intact nuclei (DAPI), expression of cytokeratins, CD45-negativity, and a diameter of 5–40 µm. In addition, EpCAM*^low/negative^* CTCs are verified by low or no expression of EpCAM. Successfully isolated cells are deposited in PCR tubes and processed for WGA prior to sequencing analysis.

### 2.2. Validation of Immunostaining on Cytospins

Prior to the establishment of the workflow, the immunostaining mastermix necessary to identify CTCs was validated on cell lines. The cell line MCF-7 was utilized as positive control for staining of cytokeratins (anti-C11/AE1/AE3-TRITC) and EpCAM (anti-vu14D9-AF488) and as negative control for CD45 (anti 3Z5S-AF647). On the contrary, leukocytes were utilized as positive control for CD45 and as negative control for cytokeratins and EpCAM. Cells were effectively stained for the expected markers (Figure S1).

### 2.3. Validation of Tumor Cell Enrichment via Parsortix™ System

First, capturing and harvesting rates within the Parsortix™ system were determined in 3 independent experiments using MCF-7 breast cancer cell line, whose diameter—measured via microscopy—was on average 18 ± 1.7 µm. In each Test 100 pre-labeled cells were spiked into healthy donor blood previously processed within the CellSearch^®^ system. In order to determine capturing rates, cells in the cassette were scanned with the fluorescence microscope integrated in the CellCelector™. Captured cells were then harvested from the cassettes into tubes with 200 µL PBS. Cell suspensions were placed on glass slides and scanned under the fluorescent microscope in order to quantify harvested tumor cells. These were identified as described above. The average capturing rate for MCF-7 cells within disposable cassettes of 6.5 µm narrow passages was of 63 ± 17.8% and out of it, 72 ± 29.6% of cells could be harvested.

### 2.4. Establishment of in Situ Staining of Captured Tumour Cells

**Figure 2:**
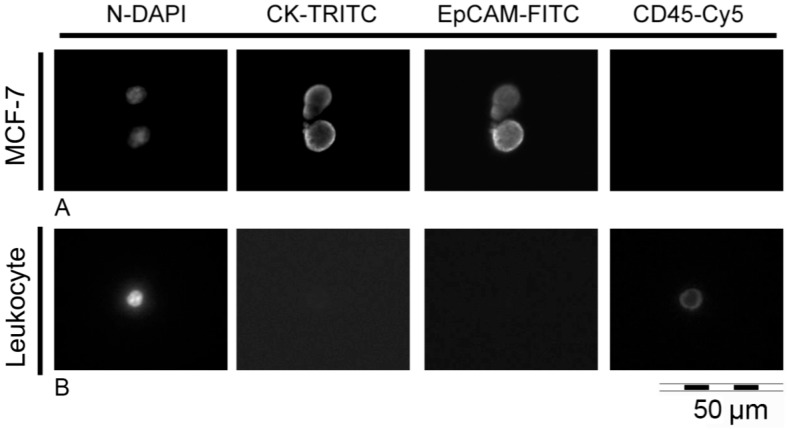
MCF-7 cells and a leukocyte labeled inside the Parsortix™ cassette. (**A**) MCF-7 cells were spiked into healthy donor blood samples and processed within the Parsortix™ system. After capturing within the cassette, tumour cells were permeabilized and stained in situ, and harvested from the system. In fluorescence microscopy MCF-7 cells exhibit signals for nucleic DNA (DAPI), cytokeratins (TRITC) and epithelial cell adhesion molecule (EpCAM-FITC); they are negative for CD45 expression (Cy5); (**B**) A leukocyte captured within the Parsortix™ system and stained in situ with the same DAPI/antibodies mastermix, shows positive staining of the nucleus (DAPI) and of CD45 (Cy5). There is no expression of cytokeratins (TRITC) and EpCAM (FITC). Magnification: 40×.

In order to optimize the workflow for clinical routine application, we subsequently established the semi-automated in situ staining of captured tumor cells. The in situ labeling approach was chosen over the staining in suspension in order to reduce the great cell loss due to pipetting procedures and/or centrifugation steps otherwise needed. Unlabeled CellSave^®^-fixed MCF7 cells were spiked into healthy donor blood samples after processing within the CellSearch^®^ system. Cells captured inside the Parsortix™ cassette were permeabilized in situ and then incubated with the antibodies/DAPI mastermix. Harvested cells were scanned under the fluorescent microscope for an intact nucleus, for cytokeratins expression as well as for EpCAM expression. A successful staining of all the selected markers could be observed (Figure 2).

### 2.5. Processing of MBC Clinical Samples: Enrichment, Detection and Isolation of EpCAM^high^ and EpCAM^low/negative^ Cells

From 07/2015 to 11/2016, 52 blood samples of 47 MBC patients were sequentially processed with our workflow. Patients´ characteristics for primary tumors and CTC fractions are reported in Table S1.

**Figure 3:**
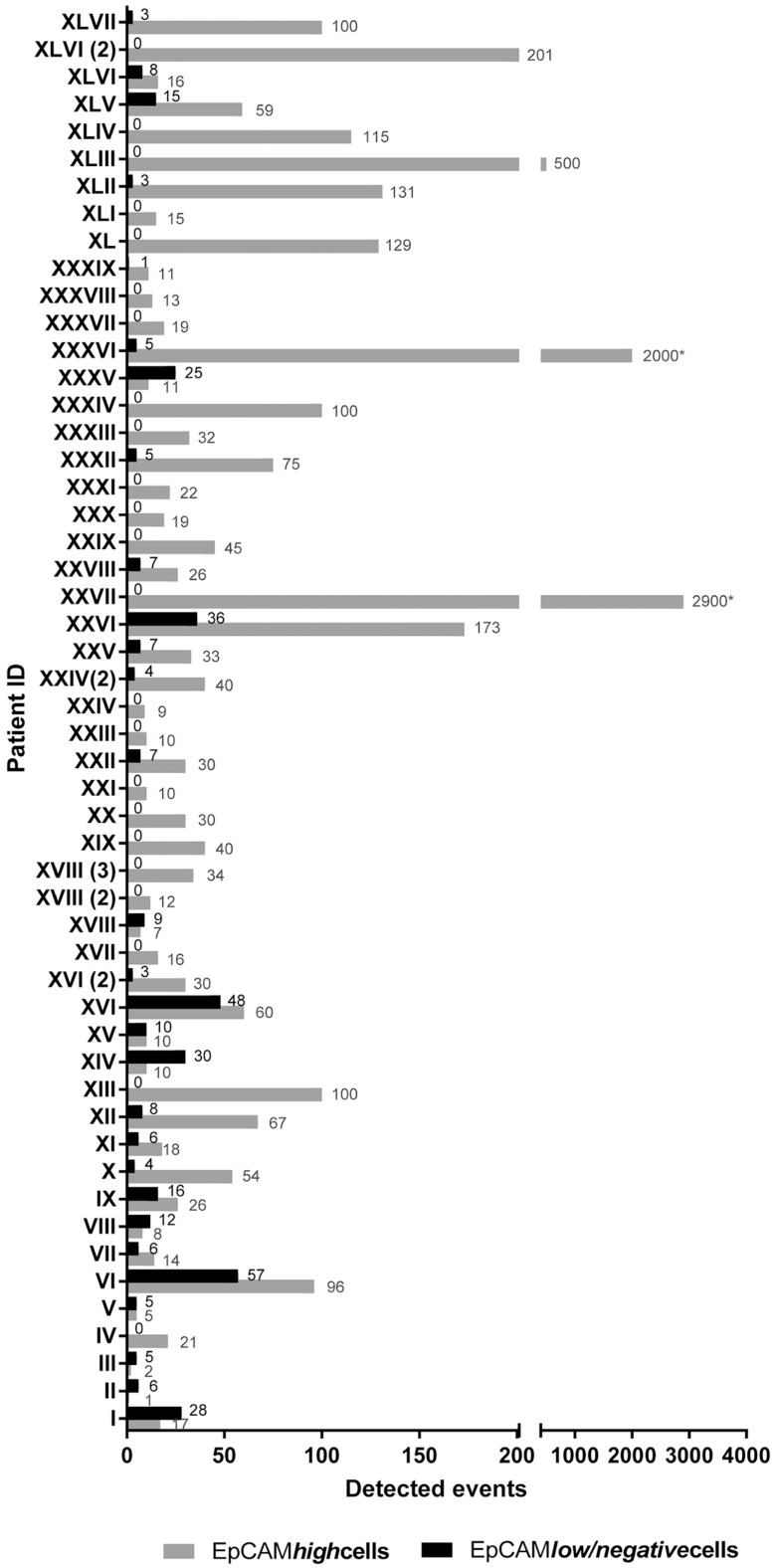
Clinical samples containing both EpCAM*^high^* and EpCAM*^low/negative^* cells. Both subpopulation of potential tumor cells were detected in 54% (28/52) of blood samples of patients enrolled in this project. No correlation in positivity rate between the two fractions of cells was observed.

In 54% (*n* = 28) of blood samples, we observed both EpCAM*^high^* and EpCAM*^low/negative^* cell subpopulations with no correlation in positivity rates (Figure 3).

**Figure 4:**
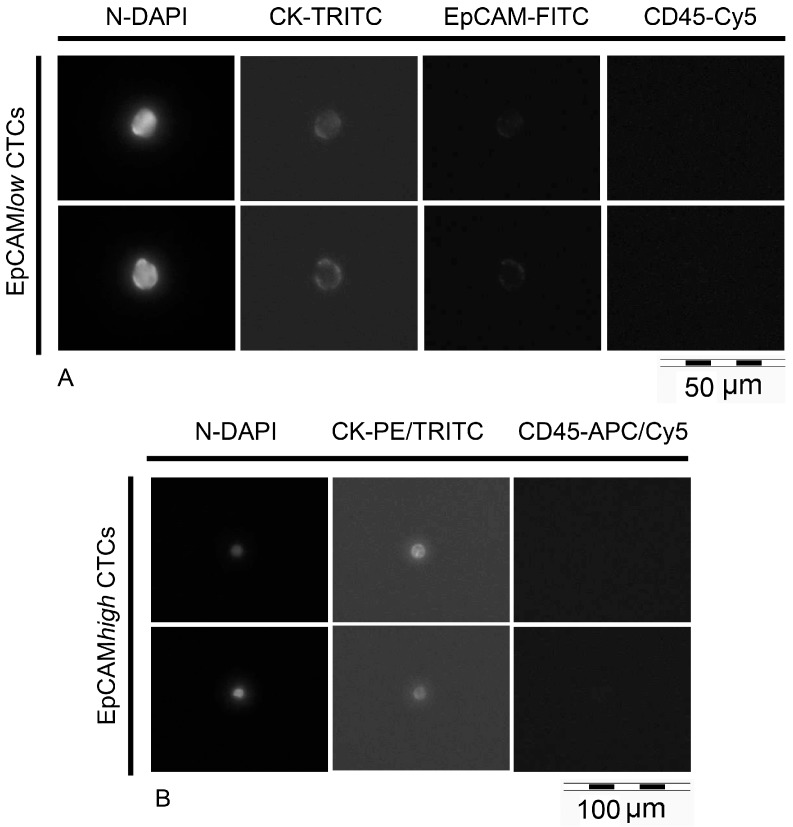
Isolated EpCAM*^low/negative^* cells and EpCAM*^high^* cells. (**A**) Prior isolation of (epithelial cell adhesion molecule ) EpCAM*^low/negative^* cells, selected events were manually scanned at a magnification of 40× in the following channels: DAPI for nucleic DNA, TRITC for cytokeratins, FITC for EpCAM, Cy5 for CD45. EpCAM*^low/negative^* cells show positive staining of nucleic DNA and cytokeratins, very weak or no staining of EpCAM, and no staining of CD45. (**B**) EpCAM*^high^* cells enriched in CellSearch^®^ cartridges were automatically scanned in the abovementioned fluorescent channels, at a magnification of 20×. They exhibit positive staining of nucleic DNA and cytokeratins, and no signals for CD45. After scanning, single cell isolation was performed in DAPI to assure the absence of contaminations.

Suitable cells for downstream analysis were selected based on their morphologic appearance and signal intensities, according to Polzer et al. [31]. We successfully isolated 107 EpCAM*^high^* and 145 EpCAM*^low/negative^* cells from 13 patients. In Figure 4, representative images of isolated EpCAM*^high^* and EpCAM*^low/negative^* cells are depicted.

### 2.6. Whole Genome Amplification of Single Isolated Cells

We tested whether enrichment and isolation conditions of our workflow were compatible with whole genome amplification in order to enable further genome analysis. We omitted the analysis of isolated cell line cells since these—in comparison to CTCs—are very robust feigning good conditions.

In order to characterize isolated EpCAM*^high^* and EpCAM*^low/negative^* cells, these were lysed and WGA was performed. Quality control PCRs for the WGA products indicated that approximately 28% of WGA libraries from EpCAM*^high^* cells show high genomic integrity compared to 8% of EpCAM*^low/negative^* cells (Table 1).

### 2.7. Mutational Analysis of PIK3CA Exons 9 and 20 in both CTC-Subpopulations

Afterwards, we investigated the status of the PIK3CA exons 9 and 20 in patient-matched EpCAM*^high^* and EpCAM*^low/negative^* CTCs. WGA products were specifically amplified for PIK3CA sequences mainly flanking hotspots E542, E545, H1047, located in exons 9 and 20. Resulting amplicons of 38 EpCAM*^high^* and of 39 EpCAM*^low/negative^* cells from 10 patients could be successfully sequenced for above reported hotspot mutations, known to cause constitutive activity of PIK3CA protein, involved in tumorigenesis [21,22].

Individual sequencing profiles of both EpCAM*^low/negative^* and EpCAM*^high^* CTCs were compared to sequencing profiles of single MCF-7—known to harbor mutations in exon 9 (amino acid change at position E542 and E545)—and of single T47D cells harboring mutations in exon 20 (amino acid change at position 1047) [33,34].

In six patients out of ten, both fractions of CTCs were classified as wild-type (WT) for the investigated PIK3CA hotspots (Table 2).

**Figure 5:**
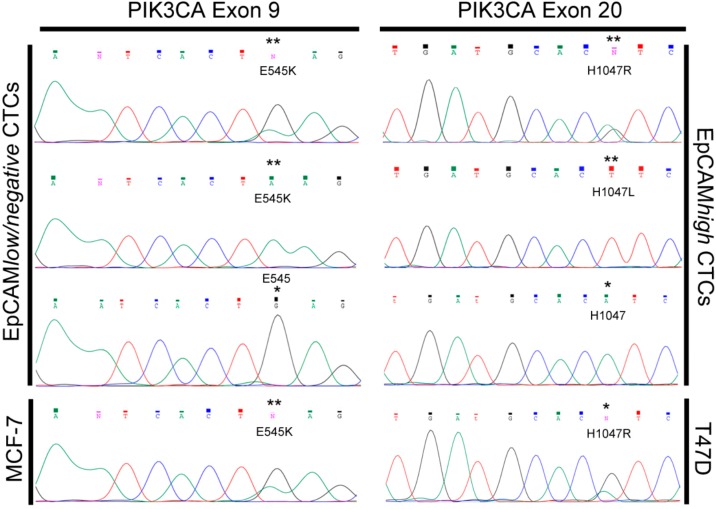
PIK3CA Exons 9 and 20 sequencing profiles. Single circulating tumor cells (CTCs) collected from four different patients and sequenced for PIK3CA exon 9 and exon 20 show Phosphatidylinositol 3-kinase catalytic subunit alpha (PIK3CA) hotspot mutations. Two EpCAM*^low/negative^* CTCs from 2 different patients exhibit the mutation 9/E545K (**) when compared to the sequencing profile of a single MCF-7 cell (**). Two EpCAM*^high^* CTCs from different patients show the mutations 20/H1047R and the 20/H1047L (**) when compared to the sequencing profile of a single T47D cell (**). CTCs recorded as PIK3CA wild-type show no mutations (*). **: base exchange; * wild-type base.

In four patients (I, IX, X, XLVII) CTCs harboring PIK3CA hotspot mutations could be observed (Figure 5) as well as tumor cells carrying the WT form of the gene.

Patient I carried PIK3CA hotspot mutations in the EpCAM*^low/negative^* CTC group only. The mutation E545K (codon 545 of the exon 9, glutamine to lysine) could be observed in one out of ten analyzed CTCs. The remaining 9 EpCAM*^low/negative^* and 2 patient-matched EpCAM*^high^* CTCs carried the WT form of the gene.

Patients X and XLVII exhibited the mutated PIK3CA in EpCAM*^high^* CTCs only. In patient X the mutation H1047R (codon 1047 of the exon 20, histidine to arginine) could be detected in two out of 5 processed CTCs. The 3 remaining EpCAM*^high^* CTCs and 1 patient-matched EpCAM*^low/negative^* CTC were classified as PIK3CA WT. In patient XLVII the rare mutation H1047L (codon 1047 of exon 20, histidine to leucine) could be detected in one out of 2 processed CTCs. The remaining EpCAM*^high^* CTC and 2 patient-matched EpCAM*^low/negative^* CTCs were classified as PIK3CA WT.

A very heterogeneous situation was observed in patient IX who carried both mutated and the WT PIK3CA in both subpopulations of CTCs. Moreover, different hotspot mutations were observed within the two different fractions of CTCs. In one out of five EpCAM*^low/negative^* CTCs, the mutation E545K was detected. Four residual EpCAM*^low/negative^* CTCs were PIK3CA WT. In two out of eleven EpCAM*^high^* CTCs the mutation H1047L (codon 1047 of exon 20, glutamine to leucine) could be observed and the remaining 9 EpCAM*^high^* CTCs were classified as PIK3CA WT.

No other base exchanges could be detected within the whole sequencing profiles of both PIK3CA exons 9 and 20.

## 3. Discussion

The clinical relevance of EpCAM*^positive^* CTCs was proved by several investigations [35,36,37]. However, whether transient and mesenchymal CTCs play a key role in the outcome of the patients is still an open question. Many studies reported the presence of EpCAM*^negative^* CTCs in the blood of patients suffering from different types of cancer. In some of these investigations the potential clinical relevance of these tumor cells was examined [3,12,14,15,16,17,38,39], often highlighting the metastatic potential of EpCAM*^negative^* CTCs [3,38,39]. In general, in all of the abovementioned studies the authors agree that this subpopulation of CTCs should be further analyzed to acquire further knowledge regarding their metastatic potential.

However, both enrichment and isolation steps for CTCs are still the bottleneck in the field of CTC analysis. Not many commercially available enrichment techniques can guarantee high cell recovery rates—regardless of protein expression—and low white blood cell contamination. Moreover, there are few technologies available to isolate single cells and most of them are time consuming, require high sample volumes, and face loss of cells.

We previously reported the existence of an EpCAM*^low/negative^* subset of CTCs in MBC patients’ samples further processed after CellSearch^®^ system [13]. Besides, we have recently described a powerful workflow to enrich EpCAM*^high^* CTCs and to micromanipulate them as single cells by combining the CellSearch^®^ system and the CellCelector™ micromanipulator [32]. Finally, in the herein described project we optimized our methods incorporating the Parsortix™ system in order to enrich, detect and isolate EpCAM*^low/negative^* tumor cells along with patient-matched EpCAM*^high^* CTCs.

The Parsortix™ system enables size-dependent enrichment of cells independently on the expression of cell surface proteins, unlike antibody dependent technologies (e.g., reported by Schneck et al. [13]). First and foremost, effective capturing and harvesting rates of the system was assessed by using cassettes with narrow passages of 6.5 µm. We observed an average of 63 ± 17.8% of captured pre-labeled MCF-7 cells and from them 72 ± 29.6% could be successfully harvested. Our data indicate slightly higher cell recovery rates than those reported by Xu et al. [40] who utilized cassettes with 10 µm narrow passages. In addition, our recovery rates are in concordance with results published by Hvichia et al. [41] who reported a range of capturing rates of 42–70% and a range of harvesting rates of 54–69% for cancer cell lines of different tumor entities (PANC1, A375, PC3, A549, T24) by utilizing 10 µm cassettes. Hence, all the data point out how recovery rates depend on both size and deformability of tumor cells, as previously already hypothesized [41].

The high capturing and harvesting rates of the Parsortix™ system are the basis for the effective CTC analysis since Neumann et al. [32] already demonstrated that single cell micromanipulation can be performed with 97% efficacy. Therefore, optimizing the in situ labeling of cells in Parsortix™ cassettes is of high importance in order to minimize cell loss, which was observed during in suspension staining procedures [40,41].

By applying the established novel workflow, we were able to successfully enrich, detect and isolate single EpCAM*^low/negative^* and patient-matched EpCAM*^high^* cells. In 54% of blood samples positive for EpCAM*^high^* CTCs also EpCAM*^low/negative^* cells could be detected without any correlation in numbers. The resulting combined CTC positivity rate is higher than that reported by de Wit et al. [12], who found both EpCAM*^high^* and EpCAM*^low/negative^* cells in 19% (5/27) of metastatic lung cancer samples. This may be explained either by the application of different CTC enrichment methods or by different abundance of EpCAM*^low^* CTCs within the different tumor entities investigated. However, in agreement to our results, de Wit et al. [12] reported a lack of correlation between numbers of both EpCAM*^high^* and EpCAM*^low/negative^* cell fractions. Of great interest for our future studies is whether EpCAM*^low/negative^* cells may provide prognostic and predictive information for early or MBC patients with no EpCAM*^high^* CTC in the CellSearch^®^ analysis.

Moreover, we proved the feasibility of our workflow for single cell downstream analysis. WGA products were of high genomic integrity in 28% of EpCAM*^high^* CTCs, accordingly with data published by Polzer et al. [31]. In contrast, the unexpected finding of 8% WGA products of high genomic integrity among EpCAM*^low/negative^* cells may point towards early apoptosis mechanisms activated in this CTC subpopulation. In future experiments, we aim to complement our staining protocol with indicators for apoptosis, a phenomenon that has was already observed in CTCs and related to patients’ outcomes in several studies [42,43,44,45,46].

In the scenario of future personalized medicine, the characterization of single CTCs besides their enumeration may also play a key role, identifying potential biomarkers (e.g., PIK3CA) to predict resistance to therapies. The status of the PIK3CA gene in MBC primary tumors as well as in CTCs is increasingly attracting attention since in patients it was often observed an increased resistance to anti-HER2 therapies caused by PI3K activating mutations [47,48]. In some reports, hotspot mutations in EpCAM*^positive^* CTCs were already described [30,31,49,50,51] and a first correlation of the PIK3CA status with the overall survival and prognosis free survival was analyzed [49].

The ability to collect and isolate different subpopulation of CTCs and to perform a single cell analysis allowed us to asses—at our best knowledge for the first time—the heterogeneity of the PIK3CA status within single EpCAM*^low/negative^* CTCs, along with patient-matched EpCAM*^high^* CTCs. We recorded the hotspot mutation E545K in EpCAM*^low/negative^* CTCs from 2 patients (patients I and IX) and the mutation H1047R in EpCAM*^high^* CTCs from another patient (patient X). These are the two most frequently reported PIK3CA hotspot mutations in breast cancer tissues (www.mycancergenome.org) as well as in EpCAM*^positive^* CTCs [30,31,49,50,51]. Interestingly, we could also detect the rare mutation H1047L in EpCAM*^high^* CTCs from two different patients (patient IX and XLVII), already described by Gasch et al. [51]. The identification of PIK3CA mutations in only EpCAM*^low/negative^* CTCs in one patient (I) and in both EpCAM*^low/negative^* and EpCAM*^high^* CTCs in another one (IX) is of high interest, suggesting that incorporating the analysis of the PI3K mutation status of EpCAM*^low/negative^* cells might be of relevance for future CTC-based therapies targeting HER2-positive CTCs as envisioned in the DETECT III study.

Since genomic analysis within single cells require prior amplification—in our workflow Ampli1 WGA and PIK3CA specific PCRs—there is the chance that base exchanges may be introduced by polymerases. However, we excluded this possibility within our sequencing results, since only specific PIK3CA hotspot mutations could be detected within the whole sequencing profiles of both exons 9 and 20.

In the future, we aim to implement our molecular analysis with the assessment of the copy number variation profiles of the EpCAM*^low/negative^* fraction of CTCs, in order to further confirm their malignancy and to investigate their clonal relatedness to the EpCAM*^high^* CTCs. In addition we will perform longitudinal follow-up analysis of the frequency of EpCAM*^low/negative^* CTCs and of the PIK3CA mutational status to investigate their evolution during treatment regimen applied in the DETECT studies.

## 4. Materials and Methods 

### 4.1. Cell Lines and Culture Conditions

For spiking and sequencing experiments, breast cancer cell lines MCF-7 and T47D were purchased from the American Type Culture Collection (ATCC, Manassas, VA, USA; catalogue numbers: HTB-22™ and HTB-133™). Cells were cultured in RPMI 1640 containing 10% fetal calf serum and 1% Penicillin-Streptomycin (all Gibco, Karlsruhe, Germany). For MCF-7, culture medium was supplemented with 25 mM HEPES (Gibco). For T47D cells 10 mM HEPES, 1 mM sodium pyruvate (Gibco) and 0.45% D-(β) Glucose solution (Sigma-Aldrich, Munich, Germany) was added. All cells were grown at 37 °C in a humidified atmosphere with 5% CO_2_. Both cell lines were authenticated via short tandem repeat (STRs) analysis. For spiking experiments, MCF-7 cells were fixed in CellSave^®^ tubes for 24 h, at room temperature (RT).

### 4.2. Patient Material

Patient blood samples were collected within the German DETECT III/IV trials (III: NCT01619111, IV: NCT02035813; for more information: www.detect-studien.de) from patients with MBC. Written informed consent was obtained from all participating patients and the studies were approved by the Ethical Committee of the Eberhard-Karls University Tuebingen (responsible for DETECT III: 525/2011AMG1; approved on the 25 October 2012) and the local Ethical Committee of the Heinrich-Heine University Duesseldorf (DETECT III: MC-531; DETECT IV: MC-LKP-668; approved on the 17 December 2013).

### 4.3. Enrichment and Enumeration of EpCAM^high^ CTCs via CellSearch^®^

The workflow starts by processing blood samples through the CellSearch^®^ system (Menarini Silicon Biosystems, Bologna, Italy) in order to enrich and enumerate EpCAM*^high^* CTCs [27]. In order to reduce the possible presence of skin contaminating cells, first milliliters of drawn blood samples are discarded and only following 7.5 mL of blood are processed through the CellSearch^®^.

After analysis, CTCs were stored in CellSearch^®^ cartridges at 4 °C in the dark and were micromanipulated within one week [32].

### 4.4. Enrichment of EpCAM^low/negative^ Cells with Parsortix™ System

Blood samples depleted of EpCAM*^high^* cells were processed within the Parsortix™ system to enrich potential EpCAM*^low/negative^* CTCs which were not captured by the EpCAM-specific ferrofluid used during the CellSearch^®^-approach.

Blood samples were pumped through a disposable cassette (Cell Separation Cassette GEN3D6.5, ANGLE plc) containing narrow passages of 6.5µm in height blocking flow-through of cells larger than most white blood cells—among them CTCs. By virtue of a priming protocol—which flushes each new cassette with ethanol 100% (*v*/*v*)—clean processing conditions could be guaranteed.

Cells captured inside the cassette were stained in situ for nucleic acid (DAPI; Roche Diagnostics GmbH, Heilingenhaus, Germany), cytokeratins (clones C11/AE1/AE3 [12,13], TRITC conjugate; Aczon Srl, Monte San Pietro BO, Italy), EpCAM (clone VU1D9 [13], Alexa Fluor^®^ 488 conjugated; Cell Signaling Technology Inc., Danvers, MA, USA) and CD45 (clone 35-ZS [13], Alexa Fluor^®^ 647 conjugated; Santa Cruz Biotechnology Inc., Dallas, TX, USA). The expression of the above reported markers (except for CD45), together with a range diameter of 4–30 µm are the only criteria available at the current state of the art, for the CTC-detection [52]. Further details about the validation of this immunostaining are provided as supplementary material.

Stained cells were harvested from the system into a PCR tube by inverting the flow direction of the buffer. In order to minimize a possible stickiness of captured and stained cells inside the cassette, PBS was supplemented with EDTA 2 mM. Samples were stored at 4 °C in the dark and micromanipulated within two days.

The Parsortix™ system was automatically cleaned with a decontaminating solution between processing of different samples.

### 4.5. Detection and Isolation of EpCAM^high^ and EpCAM^low/negative^ Cells via CellCelector™

The CellCelector™ (ALS, Jena, Germany) is a semi-automated micromanipulator consisting of an inverted fluorescent microscope (CKX41, Olympus, Tokyo, Japan) with a CCD camera system (XM10-IR, Olympus, Tokyo, Japan) and a robotic arm with a vertical glass capillary of 30 µm in diameter [53]; ALS GmbH). EpCAM*^high^* CTCs collected in CellSearch^®^ cartridges and EpCAM*^low/negative^* cells harvested from the Parsortix™ cassette were transferred onto glass slides, placed on the automatic stage of the CellCelector™ microscope and were allowed to deposit. Then, samples released from the CellSearch^®^ cartridges were automatically scanned in 20× as previously described [32]. Parsortix™ samples were manually scanned with 40× magnification using the following fluorescence channels: bright field (cell morphology), DAPI (morphology of nuclei and DNA integrity), TRITC (cytokeratins), FITC (EpCAM) and Cy5 (CD45). The following exposure times were used: 50 ms for DAPI, 300 ms (TRITC and FITC), and 500 ms (Cy5). For the analysis the CellCelector™ software 3.0 (ALS, Jena, Germany) was used. Images of single cells were stored for later documentation.

#### 4.5.1. Selection Criteria

All cells were analyzed using the following scan parameters: diameter (signals ranging from 5 to 40 µm) and grey value mean (fluorescence intensity of >2000 for cytokeratins signal). DAPI*^pos^*/Cytokeratins*^pos^*/EpCAM*^low/neg^*/CD45*^neg^* events were identified and their morphologies were checked in bright field (BF). Only events with a round shape in BF, with specific fluorescence signals in the expected fluorescent channels only and without any sign of DNA fragmentation in DAPI—pointing towards apoptosis—were selected for further isolation. Prior to cell-isolation, images were checked by two operators experienced in CTC-detection via CellSearch^®^ system.

#### 4.5.2. Cell Isolation Parameters

Cell isolation was performed in DAPI as previously described [32]. Finally, PCR tubes containing single cells in 1 µL of buffer were stored at −80 °C until further processing.

### 4.6. Whole Genome Amplification of Isolated CTCs

Genomic DNA of isolated single cells was amplified using the Ampli*1*™ WGA-Kit (whole genome amplification) according to manufacturer’s protocol (Menarini Silicon Biosystems, Bologna, Italy). This procedure is based on a ligation-mediated PCR following a site-specific DNA restriction [54]. 1 µL of WGA products was analyzed for quality control utilizing the Ampli*1*™ QC-Kit (Menarini Silicon Biosystems, Bologna, Italy), which assays four genomic areas in a multiplex PCR. As positive control high quality genomic DNA from cell lines was used. QC-PCR products were loaded on a 2% agarose-TAE gel. The presence of 3 or 4 amplicons in the QC-PCR indicates a high genomic integrity of the WGA product. Less than 3 amplicons point towards a low genomic integrity [31].

### 4.7. Sanger Sequencing of the PIK3CA Exons 9 and 20 on CTCs

All WGA-products were processed in 2 specific PCRs amplifying PIK3CA exons 9 and 20 in a volume of 25 µL containing 1 U DreamTaq DNA polymerase (Thermo Fisher Scientific Inc., Waltham, MA, USA) and 1 µL of WGA-product as template. Primers were designed—in analogy to primers used in a previous study by Hurst and colleagues [55]—to produce amplicons covering hotspot codons mainly for E542, E545 (exon 9) and H1047 (exon 20; 0.2 µM each; Table 1). They were adapted to WGA restriction sites (Mse*I*) and to exclude amplification of pseudogenes. PCR conditions were: 95 °C for 5 min, 35 cycles of 95 °C for 45 sec, 58 °C for 45 sec, 72 °C for 45 sec and finally 10 min at 72 °C. A 3% agarose-TAE gel electrophoresis was used to check for successful amplification. PCR products were purified using either the ExoSAP-IT^®^ PCR Product Cleanup (Thermo Fisher Scientific Inc., Waltham, Massachusetts, USA) or the QIAEX II^®^ Gel Extraction kit (Qiagen, Hilden, Germany) and DNA concentrations were measured with the NanoDrop™ Spectrophotometer (Thermo Fisher Scientific Inc., Waltham, MA, USA). Sanger sequencing was performed by the Genomics & Transcriptomics Laboratory (GTL) of the Biological and Medical Research Center of Düsseldorf (BMFZ) using primers designed elongating PCR primers with sequencing binding sites (Table 3).

## 5. Conclusions

In this study, we describe a robust workflow to enrich, detect and isolate patient-matched EpCAM*^high^* and EpCAM*^low/negative^* CTCs from the same clinical blood sample by combining the CellSearch^®^ system, the Parsortix™ system and the CellCelector™ micromanipulator. Furthermore, we show our workflow′s feasibility for molecular analysis and report low DNA integrity and PIK3CA mutational heterogeneity within EpCAM*^low/negative^* CTCs compared to patient-matched EpCAM*^high^* CTCs. Although our molecular data are based on a small cohort of patients, they highlight that the assessment of the PIK3CA mutational status within EpCAM*^low/negative^* CTCs along with the EpCAM*^high^* cells may provide further knowledge about resistance to HER2-targeted therapies and may help to choose optimal treatment strategies. In order to further support our findings, extended studies with larger cohorts of patients are planned.

## Figures and Tables

**Table 1:** WGA products of patients matched EpCAM*^high^* and EpCAM*^low/negative^* cells. A cohort of 145 EpCAM*^low/negative^* and 107 EpCAM*^high^* circulating tumor cells (CTCs) was processed for whole genome amplification (WGA). EpCAM*^low/negative^* CTCs show a lower genomic integrity than EpCAM*^high^* CTCs (8% vs. 28%). #: number of patients involved in the WGA of CTCs.

#	Patient ID	EpCAM*^high^* CTCs			EpCAM*^low/negative^* CTCs		
		Sorted CTCs	High Integrity WGA Products	Low Integrity WGA Products	Sorted CTCs	High Integrity WGA Products	Low INTEGRITY WGA Products
1	I	5	0	5	19	0	19
2	V	1	0	1	2	0	2
3	VI	11	5	6	24	3	21
4	VIII	8	0	8	12	0	12
5	IX	26	11	15	16	2	14
6	X	13	4	9	5	0	5
7	XI	7	3	4	6	0	6
8	XVI	9	1	8	27	1	26
9	XXIV (2)	6	1	5	4	0	4
10	XXXV	8	2	6	18	3	15
11	XLI	2	1	1	5	1	4
12	XLVI	7	2	5	4	0	4
13	XLVII	4	0	4	3	1	2
		107	30	77	145	11	134
			28%	72%		8%	92%

**Table 2:** Mutational status of PIK3CA exon 9 and exon 20 within patient-matched EpCAM*^high^* EpCAM*^low/negative^* cells. Circulating tumor cells (CTCs) characterized by the heterogeneity of phosphatidylinositol 3-kinase catalytic subunit alpha (PIK3CA) exons 9 and 20 could be detected in 4/10 patients. In the remaining patients, CTCs were classified as PIK3CA-wildtype (WT). #: number of patients involved in the WGA of CTCs.

**EpCAM*^high^* CTCs**					
#	**Patient ID**	**PIK3CA Exon 9 Mutational Analysis**		**PIK3CA Exon 20 Mutational Analysis**	
		**Sequenced CTCs**	**Mutational Status**	**Sequenced CTCs**	**Mutational Status**
1	I	2	WT	1	WT
2	VI	5	WT	2	WT
3	IX	13	WT	11	2: p.H1047L (c.CAT > CTT); 9: WT
4	X	7	WT	5	2: p.H1047R (c.CAT > CGT); 3: WT
5	XI	1	WT	3	WT
6	XVI	3	WT	3	WT
7	XXXV	1	WT	3	WT
8	XXXVI	1	WT	1	WT
9	XLVII	3	WT	3	WT
10	XLVII	2	WT	2	1: p.H1047L (c.CAT > CTT); 1: WT
		38		34	
**EpCAM*^low/negative^* CTCs**					
#	**Patient ID**	**PIK3CA Exon 9 Mutational Analysis**		**PIK3CA Exon 20 Mutational Analysis**	
		**Sequenced CTCs**	**Mutational Status**	**Sequenced CTCs**	**Mutational Status**
1	I	10	1: p.E545K (c.CAG > AAG); 9: WT	7	WT
2	VI	7	WT	7	WT
3	IX	5	1: p.E545K (c.CAG > AAG); 4: WT	3	WT
4	X	2	WT	1	WT
5	XI	1	WT	1	WT
6	XVI	0	n.d.	2	WT
7	XXXV	8	WT	8	WT
8	XXXVI	3	WT	3	WT
9	XLVII	1	WT	1	WT
10	XLVII	2	WT	2	WT
		39		35	

**Table 3:** Primer sequences for PCR amplification of exons 9 and 20 of PIK3CA and for Sanger sequencing.

PIK3CA Exon	Primer Name	Sequence (5′→3′)	Primer Length (bp)
9	forward	CATCCGATGTACCTGATTGAACTGCATGCAGACAAAGAACAGCTCAAAGCAA	52
	reverse	CATTCCTTAGATAGCTCGGAAGTCCATTGCATTTTAGCACTTACCTGTGAC	52
20	forward	CATCCGATGTACCTGATTGAACTGCATGCATTGATGACATTGCATACATTCG	52
	reverse	CATTCCTTAGATAGCTCGGAAGTCCATTGCGTGGAAGATCCAATCCATTT	50
Sequencing	forward	TCCGATGTACCTGATTGAAC	20
	reverse	TTCCTTAGATAGCTCGGAAG	20

## Supplementary Materials

Supplementary materials can be found at www.mdpi.com/1422-0067/18/9/1885/s1.

## Abbreviations

CTC	Circulating Tumor Cell
EpCAM	Epithelial Cell Adhesion Molecule
FDA	Food and Drug Administration
EMT	Epithelial to Mesenchymal Transition
MBC	Metastatic Breast Cancer
PI3K	Phosphatidylinositol 3-Kinase
PIK3CA	Phosphatidylinositol 3-Kinase Catalytic subunit Alpha
WGA	Whole Genome Amplification
WT	Wild-type
