# Supplementary material for: A Novel Workflow to Enrich and Isolate Patient-Matched EpCAMhigh and EpCAMlow/negative CTCs Enables the Comparative Characterization of the PIK3CA Status in Metastatic Breast Cancer
Source: Int J Mol Sci. 2017 Aug 31;18(9):1885. doi: 10.3390/ijms18091885 (PMC5618534; doi:10.3390/ijms18091885)
Supplement: Supplementary file 1 [file ijms-18-01885-s001.pdf]

Supplementary Materials: A Novel Workflow to Enrich and Isolate Patient-Matched EpCAM^{high} and EpCAM$^{low/negative}$ CTCs Enables the Comparative Characterization of the PIK3CA Status in Metastatic Breast Cancer

Rita Lampignano, Liwen Yang, Martin H.D. Neumann, André Franken, Tanja Fehm, Dieter Niederacher and Hans Neubauer

1. Validation of the Staining Protocol

1.1. Isolation of Leukocytes from Whole Blood

In order to isolate leukocytes from a healthy donor's blood sample, this was fixed in CellSave® tubes for 24 h, at RT, then was diluted in PBS (1:2). For the density gradient centrifugation, 15 ml of Biocoll separating solution (Merck Millipore, Billerica, MA, USA) were transferred in a new 50 mL Falcon® tube and on its top, the diluted whole blood was slowly pipetted. The solution was centrifuged at 1500 rpm, at RT, for 30 min, without breaks. Then, the upper serum phase was discarded and the interphase, including most of the leukocytes, was carefully aspirated and transferred to a new 50 mL Falcon® tube. The buffy coat was washed with 50 mL PBS and centrifuged again. Then, the supernatant was discarded and the pellet was resuspended in 1 mL PBS.

1.2. Cytospin Preparation

Prior to the establishment of the enrichment workflow, the immunofluorescence staining of markers, whose expression is required for the CTC detection within the FDA-approved CellSearch® [52], was validated on fixed MCF-7 cells and on leukocytes. At first, the cell count was determined by adding 10 µL each cell suspension to 10 µL of trypan blue (Sigma-Aldrich). Then, 10 µL of mixtures was added into two different sampling area of a Neubauer counting chamber (Paul Marienfeld GmbH & Co. KG, Lauda-Königshofen, Germany). Afterwards, an amount of 50000 cells/400 µL PBS per each suspension, was spun onto SuperFrost slides (R. Langenbrinck, Emmendligen, Germany) through a ROTOFIX 32 A centrifuge (600 g, 3 min; Hettich GmbH & Co.KG, Tuttlingen, Germany). Then, the supernatant was removed by aspiration and cytospins were left to dry overnight at RT. Afterwards, slides were stored at–20 °C.

1.3. Immunofluorescence

To validate the staining mastermix targeting nuclei, EpCAM, cytokeratins and CD45 altogether, immunostaining of fixed MCF-7 and leukocytes spun onto glass slides, was performed. At first, cytospins were washed with PBS and then incubated with 0.1% Triton X-100 (Sigma Aldrich) for 10 min, to permeabilize cell membranes. Afterwards, cells were incubated 1 h, at RT, dark, with the staining mastermix including (DAPI; Roche Diagnostics GmbH, Heilingenhaus, Germany), cytokeratins (clones C11/AE1/AE3 [12,13], TRITC conjugate; Aczon Srl, Monte San Pietro BO, Italy), EpCAM (clone VU1D9 [13], Alexa Fluor® 488 conjugated; Cell Signaling Technology Inc., Danvers, MA, USA) and CD45 (clone 35-ZS [13], Alexa Fluor® 647 conjugated; Santa Cruz Biotechnology Inc., Dallas, TX, USA), diluted in DAKO Antibody diluent (Agilent, Santa Clara, CA, USA). Cells were, then, washed twice with PBS. At the end, slides were mounted with the DAKO (Agilent) mounting medium, coverslips were applied and stored at 4 °C until imaging with the CellCelector™.

1.4. Spiking Experiments

Prior processing patients' blood samples, the method was tested on MCF-7 cells. At first, the tumor cell enrichment though Parsortix™ system (ANGLE plc, Guildford, UK)–the novel component of our workflow–was determined through three independent spiking experiments. After processing through CellSearch® system [27], three blood samples of healthy donors were spiked with a defined amount of fixed and pre-labeled MCF-7. Then, spiked samples were processed through Parsortix™ system. Capturing and harvesting rates were assessed by fluorescence imaging via CellCelector™ of both cassettes and collected cell suspension respectively.

Further spiking experiments were performed to establish the staining of tumor cells captured within Parsortix™ cassettes. Fixed MCF7 cells were spiked into healthy donor blood samples after processing within the CellSearch® system. Tumor cells which were captured inside the Parsortix™ cassette were permeabilized with 0.1% Triton X-100 for 10 min, washed with PBS and then incubated with the above reported antibodies/DAPI staining mastermix. Afterwards, stained cells were washed with PBS, harvested out of the system and the effective staining was assessed via immunofluorescence microscopy (CellCelector™).

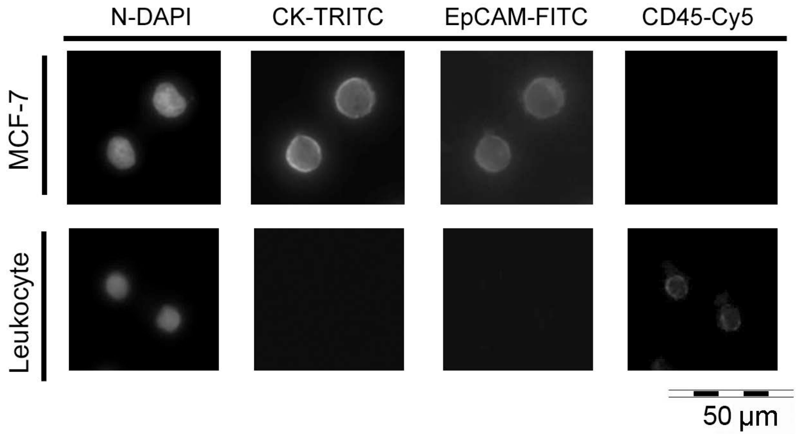

Figure S1. MCF-7 cells and leukocytes labeled on cytospins.

Table S1. Patients' characteristics for primary tumour, metastases and patient-matched EpCAM^{high} and EpCAM$^{low/negative}$ cells.

						Primary Tumour					EpCAM^{high} Cells		EpCAM$^{low/neg}$ Cells
Sample #	Patient ID	Age	HER2	ER	PR	Subtype	TNM			Grade	CellSearch Count	HER2 Status	
							T	N	M ****				
1	I	60	neg	pos	pos	lobular	T2	N0	M0	2	17	neg	28
2	II	59	*	neg	pos	*	T1c	N0	M0	3	1	neg	6
3	III	57	neg	pos	pos	ductal	T2	N1	M1	3	2	neg	5
4	IV	73	neg ***	pos ***	neg ***	*	T4	N2a	M0	2	21	pos	0
5	V	72	neg	pos	neg	ductal	T2	N1	M0	2	5	neg	5
6	VI	65	neg	pos	pos	lobular	T2	*	M0	2	96	neg	57
7	VII	60	neg	pos	pos	mucinous	T2	N1	M0	*	14	neg	6
8	VIII	62	neg	pos	neg	ductal	T2	N1	M1	3	8	*	12
9	IX	67	neg	pos	neg	ductal	T1	N0	M1	2	26	pos	16
10	X	78	neg ***	pos ***	pos ***	*	T2	N3a	M0	2	54	neg	4
11	XI	45	neg	pos	pos	ductal	T2	N0	M0	3	18	neg	6
12	XII	67	neg	pos	pos	*	T2	N1	M0	3	67	pos	8
13	XIII	50	neg	pos	pos	ductal	T1c	N0	*	3	100	pos	0
14	XIV	61	neg	pos	neg	*	T1c	N1	M1	3	10	neg	30
15	XV	47	neg	pos	pos	lobular	T1c	N1	M1	2	10	neg	10
16	XVI	73	*	neg	neg	ductal	T1c	N0	M0	3	60	neg	48
17	XVI (2)	73	*	neg	neg	ductal	T1c	N0	M0	3	30	neg	3
18	XVII	76	neg	pos	neg	lobular	T1b	N0	M0	*	16	pos	0
19	XVIII	51	neg	pos	pos	*	T1c	N0	M0	3	7	*	9
20	XVIII (2)	51	neg	pos	pos	*	T1c	N0	M0	3	12	*	0
21	XVIII (3)	51	neg	pos	pos	*	T1c	N0	M0	3	34	*	0
22	XIX	57	neg	pos	pos	ductal	T2	N0	M0	2	40	neg	0
23	XX	40	neg	pos	neg	ductal	T4d	N1	M0	2	30	neg	0
24	XXI	63	neg	pos	pos	lobular	T4b	N2a	M0	2	10	neg	0
25	XXII	60	*	pos	*	lobular	*	*	M0	*	30	pos	7
26	XXIII	57	*	pos	pos	micropapillar	T2	N3	M1	2	10	neg	0
27	XXIV	62	neg	neg	neg	ductal	T2	N0	M0	*	9	neg	0
28	XXIV(2)	62	neg	neg	neg	ductal	T2	N0	M0	*	40	neg	4
29	XXV	60	neg	pos	pos	ductal	T1c	N1	M0	2	33	neg	7

30	XXVI	56	neg	pos	pos	ductal-lobular	T2	N3a	*	2	173	pos	36
31	XXVII	74	neg	pos	pos	lobular	T2	N0	*	2	> 2900 **	*	0
32	XXVIII	62	neg ***	pos ***	pos ***	*	T1b	N0	M0	2	26	neg	7
33	XXIX	65	neg	*	*	ductal	T2	N2	M1	2	45	neg	0
34	XXX	63	neg	pos	neg	ductal	T1c	N1	M1	2	19	neg	0
35	XXXI	52	neg	pos	pos	lobular	T1	N0	M1	2	22	neg	0
36	XXXII	62	neg ***	pos ***	pos ***	*	T1b	N0	M0	2	75	neg	5
37	XXXIII	65	*	pos	pos	lobular	T2	N0	M0	2	32	pos	0
38	XXXIV	48	neg	pos	pos	ductal	Tis	N0	M0	2	100	neg	0
39	XXXV	48	neg	pos	pos	ductal	T2m	N2	M0	3	11	*	25
40	XXXVI	74	neg	pos	pos	lobular	T2	N0	M1	2	> 2000 **	pos	5
41	XXXVII	62	neg	pos	pos	lobular	T1c	N1	M0	2	19	neg	0
42	XXXVIII	63	neg	pos	neg	*	T2	N1	M0	2	13	neg	0
43	XXXIX	45	neg	pos	pos	ductal	T1c	N0	M1	1	11	neg	1
44	XL	65	neg	pos	pos	lobular	T3	N3a	M0	2	129	neg	0
45	XLI	88	*	pos	pos	lobular-ductal	T1c	N0	M0	2	15	neg	0
46	XLII	75	neg	pos	pos	lobular	T2	N0	M0	2	131	pos	3
47	XLIII	67	*	pos	pos	*	T2	N1	M0	2	500	neg	0
48	XLIV	54	neg ***	pos ***	pos ***	*	T1	N0	M0	2	115	neg	0
49	XLV	65	neg	pos	pos	ductal	T1c	N1	M0	3	59	neg	15
50	XLVI	48	neg	pos	pos	ductal	T1c	N0	M0	2	16	pos	8
51	XLVI (2)	48	neg	pos	pos	ductal	T1a	N0	M0	2	201	pos	0
52	XLVII	51	neg	pos	pos	ductal	T4b	N3a	M0	3	100	neg	3

* Unknown; ** CTC count could not be determined; *** receptor status determined on the DCIS; **** M status of primary tumour at the time of diagnosis. #: amount of processed blood samples.
